# Supplementary material for: The interactive influence of gender and ergonomic factors, alongside psychosocial associations, on work-related musculoskeletal disorders in Saudi dental students: a cross-sectional study
Source: PeerJ. 2025 Aug 14;13:e19798. doi: 10.7717/peerj.19798 (PMC12358109; doi:10.7717/peerj.19798)
Supplement: Supplemental Information 2 [file peerj-13-19798-s002.docx]

**Title and Abstract**

- **Title (#1a)**: Clearly indicates the study design as a cross-sectional study.
- **Abstract (#1b)**: Summarizes the background, objectives, methodology, and main findings.

**Introduction**

- **Background/Rationale (#2)**: The introduction provides a detailed background on WMSDs, their implications, and their association with training hours per week, academic level, gender, stress, and social support.
- **Objectives (#3)**: The study aims to explore the interactive effects of gender, with ergonomic factors namely training hours assigned per week and academic levels . In addition, the association of stress and social support with WMSDs among dental students.

**Methods**

- **Study Design (4)**: Described as an analytical cross-sectional study.
- **Setting (#5)**: Conducted at a dental school in Western Saudi Arabia; participants were undergraduate students across all academic levels.
- **Eligibility Criteria (6a)**: Inclusion and exclusion criteria are clearly defined (e.g., interns, pregnant students, and those with prior musculoskeletal injuries were excluded).
- **Outcomes, Exposures, Predictors (#7)**: Clearly defined outcomes (WMSDs), exposures (gender, stress, social support), and predictors (academic level, training hours).
- **Data Sources/Measurement (8)**: Described validated tools like the validate NMQ, MSPSS, and PSS-10 for data collection.
- **Bias (9)**: Acknowledges potential recall bias and social desirability associated with self-reported data.
- **Study Size (10)**: Sample size of 409 participants with a response rate of 52%.
- **Quantitative Variables (#11)**: Quantitative variables like age and training hours were analyzed; median and interquartile ranges are reported.
- **Statistical Methods (#12a-e)**: Describes statistical methods used, including logistic regression and interaction analysis, with measures to address multicollinearity (mean centered of training hours per week).

**Results**

- **Participants (#13a-c)**: Reports on recruitment, participation rates, and demographic details.
- **Descriptive Data (#14a-b)**: Provides characteristics of participants, including demographics and academic level, with stratified results for WMSDs.
- **Outcome Data (#15)**: The study reports prevalence rates and body regional distribution of WMSDs.
- **Main Results (#16a-c)**: Includes adjusted odds ratios with confidence intervals, highlighting significant associations of WMSDs with e.g., gender, academic level and assigned training hours per week interactions).
- **Other Analyses (#17)**: descriptive analyses of sample characteristics and body regional distribution of WMSDs.

**Discussion**

- **Key Results (#18)**: Summarizes the main findings, emphasizing the significant role of gender and ergonomic factors.
- **Limitations (#19)**: Discusses limitations, such as self-reported data, response rates, and the cross-sectional design's inability to infer causality.
- **Interpretation (#20)**: Interprets findings cautiously, comparing them with previous studies.
- **Generalisability (#21)**: Discusses the limited generalizability due to the study's single-institution sample.

**Other Information**

- **Funding (#22)**: Declares no external funding.
- **Ethical Approval**: Approved by the Research Ethics Committee of the College of Dentistry, Taibah University.
- **Acknowledgments**: Thanks undergraduate students who participated in the study.
- **Conflicts of Interest**: Authors declare no conflicts of interest.
